# Adult picky eaters with symptoms of avoidant/restrictive food intake disorder: comparable distress and comorbidity but different eating behaviors compared to those with disordered eating symptoms

**DOI:** 10.1186/s40337-016-0110-6

**Published:** 2016-10-29

**Authors:** Hana F. Zickgraf, Martin E. Franklin, Paul Rozin

**Affiliations:** 1 Department of Psychology, University of Pennsylvania, 3720 Walnut Street, Philadelphia, PA 19104 USA; 2 Department of Psychiatry, University of Pennsylvania Perelman School of Medicine, 3535 Market Street, Philadelphia, PA 19104 USA

**Keywords:** Avoidant/Restrictive Food Intake Disorder, ARFID, Picky eating, Selective eating, Restrictive eating, EAT-26

## Abstract

**Background:**

One presentation of Avoidant/Restrictive Food Intake Disorder (ARFID) is characterized by picky eating, i.e., selective eating based on the sensory properties of food. The present study has two aims. The first is to describe distress and impairment in individuals with ARFID secondary to picky eating. The second is to determine whether eating behaviors hypothesized to be specific to picky eating can differentiate picky eaters with and without ARFID from typical eaters (e.g., individuals not reporting picky or disordered eating) and individuals who strongly endorse attitudes associated with anorexia and bulimia (eating disordered attitudes).

**Methods:**

Participants were recruited from Amazon’s Mechanical Turk (*N =* 325) and an online support group for adult picky eaters (*N =* 81). Participants were grouped based on endorsement of picky eating, ARFID symptoms, and elevated eating disordered attitudes on the Eating Attitudes Test (EAT-26). The resulting four eating behavior groups were compared on measures of distress and impairment (e.g., anxiety/depression and, obsessive compulsive disorder symptoms, eating-related quality of life) and on measures of eating behaviors associated with picky eating (e.g., food neophobia, inflexibility about preparation and presentation of preferred foods, sensitivity to sensory stimuli, and eating from a very narrow range of foods). The groups were compared using one way ANOVA with post-hoc Tamhane’s T2 tests.

**Results:**

On measures of distress and impairment, participants with ARFID reported higher scores than both typical eaters and picky eaters without ARFID, and comparable scores to those with disordered eating attitudes. Three of four measures of picky eating behavior, eating inflexibility, food neophobia, and eating from a range of 20 or fewer foods, distinguished picky eaters with and without ARFID form typical eaters and those with disordered eating attitudes. Picky eaters with ARFID reported greater food neophobia and eating inflexibility, and were more likely to eat from a narrow range of foods, compared to picky eaters without ARFID.

**Conclusions:**

Adult picky eaters can be differentiated from those with symptoms of anorexia and bulimia by their stronger endorsement of food neophobia and inflexible eating behaviors, and by eating from a very narrow range of foods. Picky eaters with ARFID symptoms can be differentiated from picky eaters without these symptoms on the basis of these three eating behaviors, and by their higher endorsement of internalizing distress, OCD symptoms, and eating-related quality of life impairment. This study provides evidence that ARFID symptoms exist independently of symptoms of other eating disorders and are characterized by several distinct eating behaviors. In a clinical analogue sample of disordered eaters, ARFID symptoms were associated with distress and impairment at levels comparable to symptoms of anorexia and bulimia.

## Plain english summary

Picky eaters are people who avoid many new and familiar foods because they dislike their taste, smell, texture, or appearance. When it is severe, picky eating can lead to weight loss or difficulty maintaining a healthy weight, nutritional deficiencies, dependence on supplements to get adequate nutrition or calories, or difficulty engaging in daily life because of shame, anxiety, or inconvenience. People who experience one or more of these consequences because of their picky eating can be diagnosed with Avoidant/Restrictive Food Intake Disorder (ARFID). People who restrict the amount of food they consume because they are afraid of gaining weight or being fat (and who usually engage in excessive exercise or purging behaviors to get rid of calories) are diagnosed with anorexia or bulimia when their eating leads to weight loss, nutritional problems, or interferes with life. ARFID is a new diagnosis, and in this paper, we show that 1) adults with ARFID symptoms are just as distressed, and just as likely to have symptoms of depression, anxiety, and obsessive compulsive disorder, as those with anorexia or bulimia, but that 2) adults with ARFID symptoms show very different types of eating behavior from adults with symptoms of anorexia or bulimia.

## Background

Picky eating is characterized by eating from a narrow range of accepted foods, rigidity about the preparation and presentation of preferred foods, and unwillingness to try new foods [32]. Picky eating appears to be quite common across the lifespan, although prevalence estimates vary widely (e.g., 5.6–56 %), likely because of the lack of a standardized, widely used instrument to measure picky eating behavior [32]. Although in most cases, picky eating does not interfere with weight status, growth, or psychosocial functioning, severe picky eating can lead to symptoms of avoidant restrictive food intake disorder (ARFID; [2, 36]). ARFID can be diagnosed in individuals of any age or developmental level whose restrictive eating leads to weight loss, nutritional deficiencies, dependence on nutritional supplementation or enteral feeding, or psychosocial impairment, and cannot be attributed entirely to shape and weight concerns or medical comorbidity [2]. Although one presentation of ARFID is described as “manifesting behaviorally as picky eating,” to our knowledge there has not been a direct examination of the relationship between picky eating behavior and ARFID symptoms ([2], p. 335).

There is some evidence from the picky eating literature for an association between picky eating and symptoms of ARFID. Picky eating in children younger than five is a risk factor for underweight or poor growth (e.g., [3, 8, 9]). Picky eating has been associated with lower fruit and vegetable consumption in school aged children [14] and with malnutrition in the elderly [21], and has been nominated as a barrier to healthy eating by several focus group samples (e.g., [19, 33]). Childhood picky eating has been associated with family stress and mealtime conflict (e.g., [17, 22]), and in one online survey, adult picky eaters reported higher rates of eating-related quality of life impairment and eating-specific social anxiety compared to typically-eating peers [34].

Picky eating is a common behavioral variant, affecting a large minority of healthy adults and children. Although there are consistent reports of associations between picky eating and symptoms of ARFID, it is likely that only more severe picky eating is strongly associated with these outcomes, whereas less severe picky eating is relatively benign. However, as yet there are few validated measures of picky eating, and research on markers or measures of picky eating severity is in its very early stages. In addition, most of the literature linking picky eating behavior to outcomes consistent with ARFID symptoms was conducted prior to the publication of DSM-5, the first edition to include the ARFID diagnosis, and therefore does not directly assess ARFID symptoms.

The present study is the first to explore ARFID symptoms secondary to picky eating in adults. This study has two aims. The first is to describe symptoms of internalizing psychopathology, obsessive compulsive disorder, and reduced eating-related quality of life in individuals with ARFID secondary to picky eating. The second aim is to determine whether eating behaviors hypothesized to be specific to picky eating can differentiate picky eaters with and without ARFID from typical eaters and individuals who strongly endorse attitudes associated with anorexia and bulimia. In order to explore these aims, participants were divided into four eating behavior categories on the basis of their self-reported eating attitudes and behaviors: picky eaters with ARFID symptoms secondary to their picky eating, picky eaters without ARFID symptoms, individuals reporting attitudes and behaviors associated with anorexia or bulimia (e.g., shape and weight concerns, dieting behaviors, fear of fatness; referred to throughout this manuscript as “disordered eating attitudes”), and participants reporting neither picky eating nor disordered eating attitudes (referred to as “typical eaters”).

There is some symptom overlap between ARFID and typical and atypical anorexia nervosa. The consequences of the eating disturbance (e.g., weight loss and under-nutrition) are the same, although the severity threshold is higher for typical anorexia. The nature of the eating disturbance (e.g., restriction due to unrealistic shape and body weight concerns vs. restriction due to aversion to the sensory properties of food) distinguishes the two disorders [2]. Prior to the introduction of the ARFID diagnosis, individuals with ARFID were often referred to treatment designed for individuals with disordered eating attitudes consistent with restricting anorexia [23]. In a series of retrospective chart reviews up to 2014, 12–22 % of adolescents referred to eating disorder clinics meet criteria for ARFID rather than for a restricting eating disorder associated with weight and shape concerns and body image disturbance (e.g., [23, 24]). In order to show that ARFID is distinct from other restricting eating disorders, it is necessary to show that individuals with self-reported ARFID symptoms can be differentiated from those with self-reported attitudes associated with anorexia and bulimia on the basis of either comorbidity or eating behavior. In addition, because ARFID is a new diagnosis, this investigation is the first to show that an analogue clinical sample with ARFID shows comparable distress and impairment to a more established analogue clinical sample (e.g., risk for eating disorders based on self-report).

In childhood, picky eating has been concurrently and prospectively associated with internalizing psychopathology [17, 22, 32, 36]. Adult picky eaters score higher than peers on measures of depression and obsessive compulsive disorder symptom severity, and report lower eating-related quality of life [18, 34]. Because it is likely that only the most severe picky eating is actually impairing, we hypothesize that picky eaters with ARFID symptoms will report more OCD and internalizing symptoms, and greater eating related quality of life impairment than picky eaters without these symptoms. Consistent with the existing literature on disordered eating symptoms, we predict that participants who report disordered eating attitudes will also report higher levels of psychopathology and eating related quality of life impairment than picky eaters and participants who did not report any eating disturbance (e.g., Wildes et al. [34]).

Picky eaters across the lifespan eat from a narrow range of preferred foods (e.g., fewer than 20 different foods), refuse to try new foods (e.g., neophobia), are rigid and particular about the ways in which preferred foods are prepared or presented, and report heightened sensitivity to sensation across sensory modalities (e.g., [6, 7, 10, 14, 17, 18, 22, 30, 34]). Severity of picky eating has yet to be behaviorally defined; if picky eaters who report more neophobia, dietary rigidity, and sensory sensitivity, and eat from a narrow range of foods, are more likely to also report ARFID symptoms, this supports the hypothesis that more pronounced picky eating behavior is a marker of picky eating severity and risk for ARFID. In the present study, picky eaters with and without ARFID are compared to participants who report disordered eating attitudes and to typical eaters, on measures of food neophobia, eating inflexibility, sensory sensitivity, and eating from a narrow range of foods (fewer than 20 individual foods; e.g., [18]). We hypothesize that these variables will distinguish picky eaters from typical eaters and those with disordered eating attitudes, and participants with ARFID symptoms less severe picky eaters.

## Methods

### Participants

Two samples were recruited for this study. One sample (*N =* 332) was recruited from Amazon’s Mechanical Turk (MTurk), a website where workers are paid small sums of money to complete surveys. All MTurk participants were living in the United States and spoke English fluently. Although they are not representative of the US population, MTurk workers in the US are representative of the internet-using US population in terms of geographical location, race/ethnicity, education attainment, and occupation [5, 25]. MTurk workers (and the population of internet users of which they are relatively representative) differ systematically from the general US population in that they tend to be younger, better educated, and underemployed [25, 28]. The second sample (*N =* 81) was recruited from an online support group for adult picky eaters (Picky Eating Adults Support; PEAS). These participants were recruited via several posts to the message board explaining the purpose of the research and given the option to be entered into a raffle to win one of four $25 Amazon.com gift cards after participating.

### Measures

#### Demographics

Participants reported their ages and responded to two items assessing race/ethnicity and education attainment (Table 2).

**Table 1:** Eating behavior classification by sample

	Full sample	Mechanical Turk	Mechanical Turk picky eaters	Support group picky eaters
Typical eater	46.6 %	58.2 %	NA	NA
Picky eater only	32.8	27.1	82.2 %	55.6 %
Eating disordered attitudes	9.4	11.7	8.4	0
ARFID	11.3	3.1	9.3	44.4
*N*	406	325	107	81

#### Picky eating

The picky vs. non-picky samples were selected based on responses to a single item: “I am a picky eater (e.g., I dislike many foods that most people eat, or I am particular about how my food is prepared/served).” As Taylor and colleagues note in their recent review of the picky eating literature (2015), there is no single agreed-upon measure of picky eating. However, agreement with this single selection item has been used to identify picky eaters in both childhood (e.g., [17, 22]) and adulthood (e.g., [18, 34]). Some of these authors used a True/False response scale, whereas others selected picky and non-picky eaters based on extreme responses on a Likert scale. In the present study, we chose to use a broad selection criterion to identify picky eaters, as did Kauer et al. [18]) in one of the only previous studies of adult picky eating. Because our second study aim involves identifying measures of picky eating severity that might differentiate relatively unimpaired picky eaters from those reporting ARFID symptoms, we chose this broad selection criterion in order to better sample the full range of picky eating behaviors. Participants who agreed with the statement “I am a picky eater” by responding 3–5 (e.g., “slightly agree,” “agree,” or “strongly agree”) on a 0–5 Likert-type scale were classified as picky eaters, and those who responded 0–2 (e.g., “strongly disagree,” disagree,” or “slightly disagree”) were classified as non-picky.

#### ARFID symptoms

Participants who reported that they were picky eaters responded to an author-developed questionnaire assessing the presence of the four criterion-A symptoms of DSM-5 ARFID: weight loss or difficulty maintaining weight, nutritional deficiency, dependence on nutritional supplementation, or psychosocial impairment (adapted from the Structured Clinical Interview for DSM-5; [12]). See Appendix for the full text of this measure. Participants who indicated that at least one ARFID symptom was present to “a significant” degree were classified as either ARFID-only or comorbid eaters, depending on their scores on the screening instrument for disordered eating. A count variable represented the total number of significant symptoms endorsed.

#### Eating-related quality of life

The Clinical Impairment Assessment questionnaire is a measure designed to assess the quality of life impacts of eating, over-exercising, and weight and shape concerns [4]. Wildes et al.[34])) modified this instrument to make it specific to the consequences of eating. Picky eating behaviors were not mentioned; the questions were asked about “eating habits” in general. Participants responded on a 4-point Likert scale (not at all – a lot) to 16 items assessing various consequences of eating behaviors over the past month, including “…made it difficult to concentrate,” “…made you feel critical of yourself,” “…stopped you from going out with others,” and “…affected your work or school performance.” Internal consistency was very high: *α* = .95.

#### Disordered eating attitudes

The Eating Attitudes Test-26 (EAT-26) is a validated screening tool for attitudes associated with anorexia and bulimia. The EAT-26 assesses concern with shape and weight, fear of fatness, perception of societal pressure to be thin, and binging, compensatory, and restricting behaviors. The EAT-26 uses a 1–6 Likert-type response scale and is scored by recoding responses such that scores of 1, 2, or 3 = 0 and 4 = 1, 5 = 2, and 6 = 3. The resulting scores are summed. A score of 20 or higher is considered potentially indicative of disordered eating attitudes and behaviors [15]. Internal consistency of this measure was high: *α* = .88. Participants also responded to nine items from the Eating Disorder Diagnostic Scale [31] assessing the intensity of weight and shape-related cognitions and the frequency of restricting and compensatory behaviors. These items were summed to create a continuous score. Internal consistency for these nine items was good: *α* = .79. The EAT-26 is more sensitive to restrictive eating behaviors and attitudes than to binge eating; we chose to use this measure because our goal was to isolate the impact of picky eating on weight, nutritional status, and psychosocial functioning by identifying and separating individuals with restricting disordered eating, which leads to similar symptoms.

#### Internalizing distress

The 21-item version of the Depression, Stress, and Anxiety Scale (DASS-21) was used as a measure of general internalizing distress. The DASS-21 includes items assessing the degree to which participants have felt negative emotions during the past week. Although the scale has three factors, these factors were highly correlated in the present samples: *r* > .57. The 21 items were summed to create a single variable assessing current internalizing distress [16]. Internal consistency for the full scale was excellent: *α* = .95.

#### Obsessive compulsive disorder symptoms

The Obsessive Compulsive Inventory-Revised (OCI-R) is an 18-item instrument that measures distress/disturbance from six categories of obsessive compulsive behaviors: hoarding, ordering, checking, washing, obsessing, and neutralizing. The instrument can be summed to create a single variable assessing the severity of OCD symptoms. The instrument has been widely used in non-clinical populations, and a cut-off score of 21/72 has been shown to differentiate individuals with OCD from healthy and anxious controls [11]. The 18 items of the OCI-R had excellent internal consistency: *α =* .92.

#### Narrow range

All participants were asked whether they “only eat from a very narrow range of foods (fewer than 20 different individual foods).” This item was taken from Kauer et al. [18]) survey; in that sample, a much greater proportion of picky than non-picky eaters endorsed this behavior.

#### Food neophobia

All participants responded to the Food Neophobia Scale (FNS), a 10-item scale measuring reluctance or unwillingness to try new foods [26]. Participants used a 6-point Likert scale to respond to items such as “I don’t trust new foods,” “I am constantly sampling new and different foods (reversed)”and “If I don’t know what is in a food, I won’t try it.” The FNS has been shown to predict eating behavior in the laboratory (e.g., [26]). Internal consistency in this sample was high: *α* = .96.

#### Inflexible eating behavior

There is no existing instrument to measure the rigid eating behavior associated with picky eating severity. We developed the Inflexibility Index based on items from Kauer et al. [18]) more exhaustive survey of eating behaviors that reflect inflexibility in accepting non-preferred foods and rigidity around the preparation and preparation of preferred foods (e.g., “the thought of eating a food I do not like fills me with anxiety; “I avoid letting different foods touch on my plate, even when they are both foods that I like”). See Appendix for the full text of this measure. Participants responded to 12 items on a 0–5 Likert-type scale, and responses to the 12 items were summed to create scores ranging from 0–60. Unrotated principle components analysis (PCA) indicated that in the full sample, all items loaded on a single factor, with loadings > .50. Internal reliability for all 12 items was excellent: *α* = .92. See Appendix for the full text of this measure.

#### Sensory sensitivity

All participants responded to an author-developed 11-item scale measuring over-responsivity to taste, texture, smell, and sound. Items were based on other instruments designed to measure self-reported sensory functioning in adults, including the SensOR Assessment [29] and the Glasgow Sensory Questionnaire [27]. See Appendix for the full text of this measure. An unrotated PCA in the full MTurk sample indicated that all 11 items loaded on a single factor with loadings > .50. The scale was internally reliable: *α* = .82.

### Procedures

Participants responded to all study instruments in a single online survey. Participation took 20–60 min. For both samples, two attention check questions (e.g., and two questions designed to identify bots (by asking participants to select which of four grammatically correct sentences did not make sense, e.g., “pigs eat red and anger”) were used to ensure the quality of the data. All participants who consented to participate passed both the bot and attention checks. All study instruments and procedures were approved for human subjects by the Institutional Review Board of the University of Pennsylvania, and all participants provided informed consent prior to participating.

#### Group assignment

In order to address the potential overlap between symptoms of ARFID due to picky eating (weight loss, nutritional deficiency/supplement dependence, and psychosocial impairment) and potential consequences of the restrictive eating attitudes assessed by the EAT-26, participants were separated into four eating behavior categories: typical eating, picky eating only, disordered eating attitudes only, and ARFID symptoms only. Group assignment was based on self-described picky eating, endorsement of ARFID symptoms and score on the EAT-26.

Participants who agreed with the statement “I am a picky eater” also responded to questions assessing ARFID symptoms. All participants responded to the EAT-26. Participants who were not self-reported picky eaters were classified based on their response to the EAT-26 as either typical eaters or, if they scored a 20 or greater on the scale, as having disordered eating attitudes. Self-identified picky eaters who did not endorse any significant ARFID symptoms and scored lower than 20 on the EAT-26 were classified as picky eaters; those who endorsed ARFID symptoms and scored lower than 20 on the EAT-26 were classified as “ARFID only,” and participants who scored 20 or greater on the EAT-26 and did not endorse significant ARFID symptoms were classified as having disordered eating attitudes. See Table 1 for eating behavior group classifications.

**Table 2:** Sample descriptives: Age, race, education attainment

		Mechanical Turk	Support group
Age mean (SD)		33.92 (10.54)	40.42 (13.31)
Education attainment N (%)	High school	41 (13.6 %)	5 (6.2 %)
	Some college	73 (26.1)	19 (23.5)
	2 year degree	44 (13.5)	11 (13.6)
	4 year degree	88 (27.1)	33 (40.7)
	Advanced degree	34 (10.4)	11 (13.5)
Race/ethnicity N (%)	African American	20 (6.2 %)	2 (2.5 %)
	East Asian	12 (3.7)	0
	Hispanic	12 (3.7)	0
	Multiracial	16 (4.9)	5 (6.2 %)
	Native American	1 (0.3)	0
	Southeast Asian	8 (2.5)	0
	White	254 (78.2)	74 (91.4)
Total N		325	81

### Data analysis

One-way ANOVAs with Tamhane’s T2 post-hoc comparisons (with no assumption of equal group size or homogeneity of variance) with listwise deletion for cases with missing data, were conducted to compare the four groups on all continuous dependent variables (Aim 1: eating-related quality of life, internalizing distress, disordered eating attitudes, OCD symptomatology; Aim 2: food neophobia, eating inflexibility, sensory sensitivity). A chi-square analysis with four degrees of freedom was conducted to compare the groups on proportion eating from a range of 20 or fewer foods; in order to minimize Type II error, the only post-hoc comparisons were made between picky eaters with ARFID and those without, and picky eaters without ARFID and participants endorsing eating disordered attitudes.

## Results

### Sample descriptives

Support group members were older than MTurk participants (M_MTurk_ = 33.9 (10.54), M_PEAS_ = 40.4 (13.3), *t*(106.3) = 4.09, *p* < .001, *d* = 0.54) and a higher proportion of the support group sample was female (50.5 % vs. 71.3 %, *χ* ^2^(1) = 11.89, *p* = .001, *r* = .17). Participants self-reported their education attainment. For both the MTurk and PEAS groups, the modal education attainment was a 4-year college degree (see Table 2 for age and education attainment).

Both samples were more than 70 % White. A significantly higher proportion of the PEAS sample was White vs. other racial groups, compared to the MTurk sample: *χ* ^2^(1) = 7.07, *p* = .01, *r* = .13 (see Table 2 for race/ethnicity sample descriptives).

The proportion of women vs. men was higher in both the ARFID and disordered eating attitude categories; men and women were more equally represented in both the typical and picky eater categories (Table 3). One participant in the MTurk sample and one participant in the support group sample selected the “other” option for gender; the MTurk participant was a typical eater and the support group participant was a picky eater.

**Table 3:** Proportion of women in eating behavior categories

	Full sample		Mechanical Turk sample	
	*n*	% female	*n*	% female
Typical eater	189	45.0	188	45.0
Picky eater only	133	54.9	88	48.9
Eating disordered attitudes	38	76.3	38	76.3
ARFID	45	75.6	10	70.0

### DSM-5 ARFID criteria

Self-identified picky eating was relatively common in the MTurk sample; 33 % of participants (*n =* 107) agreed with the statement “I am a picky eater.” All support group participants were self-identified picky eaters. Two self-described picky eaters from this sample did not respond to the ARFID items; they were deleted from subsequent analyses.

Compared to the support group, picky eaters in the MTurk sample endorsed significantly fewer symptoms of ARFID. Mean significant symptom endorsement in the picky MTurk sample was 0.12 (0.47) compared to 0.82 (1.02) in the support group sample; *t*(102.41) = 5.67, *d* = 2.06, *p* < .001. See Table 4 for the percentage of participants from each sample endorsing each ARFID symptom.

**Table 4:** Percent of self-identified picky eaters endorsing ARFID symptoms

	Mechanical Turk picky eaters (*n* = 105)			Support Group (*N* =79)		
	None	Some	Significant	None	Some	Significant
Weight loss	72.0 % (77)	23.4 % (25)	2.8 % (3)	86.1 % (68)	12.7 % (10)	1.3 % (1)
Nutritional deficiencies	65.4 (70)	31.8 (34)	0.9 (1)	34.2 (27)	60.8 (48)	5.1 (4)
Dependence on nutritional supplements	63.6 (68)	28.0 (30)	5.6 (6)	31.6 (25)	53.2 (42)	15.2 (12)
Occupational interference	86.9 (93)	11.2 (12)	0	57.0 (45)	34.2 (27)	8.9 (7)
Social interference	82.2 (88)	15.0 (16)	0.9 (1)	15.2 (12)	53.2 (42)	31.6 (25)
Family interference	83.2 (89)	13.1 (14)	1.9 (2)	35.4 (28)	44.3 (35)	20.3 (16)

### Study aim 1: eating disturbance and comorbidity

One-way ANOVAs indicated that the four eating behavior groups differed significantly on all four variables. As hypothesized, participants endorsing eating disordered attitudes on the EAT-26 had significantly higher scores on a separate measure, the self-report version of the Eating Disorder Diagnostic Scale, compared to all other groups (*p* < .001). Picky eaters did not differ significantly from either typical eaters or participants with ARFID on this measure; however, participants with ARFID scored higher on this measure compared to typical eaters (*p* < .03).

Similar patterns emerged in the post-hoc comparisons for the measures of internalizing distress and OCD symptoms. Typical eaters scored significantly lower on each measure than any other group (*p* < .001). Picky eaters reported lower scores than participants eating disordered attitudes (*p* < .02), and, at a trend level, than participants with ARFID (*p* = .054 for internalizing distress and *p* = .053 for OCD symptoms). Participants with ARFID and those with eating disordered attitudes did not differ from each other on either measure.

A different pattern of group differences emerged for scores on eating-related quality of life impairment. On this variable, typical eaters scored lower than any other group (*p* < .001 for comparisons with ARFID and eating disordered attitude groups; *p* = .004 for comparison with picky eaters); picky eaters scored significantly higher than typical eaters and significantly lower than participants with ARFID or eating disordered attitudes (*p* < .001), and participants with ARFID symptoms and eating disordered attitudes did not differ from one another. See Table 5 for means group means and *F*-statistics for these analyses.

**Table 5:** Group differences: eating-related quality of life and psychopathology variables

Eating behavior classification	*n*	Mean (SD)	EAT-26 (0–40)	EDDS (0–9)	EQoL (0–5)	DASS-21 (0–57)	OCI-R (0–72)
Typical eater	189		5.71_a_ (4.83)	2.49_a_ (1.94)	0.24_a_ (0.36)	9.96_a_ (10.86)	9.61_a_ (10.01)
Picky eater	128		5.30_a_ (5.57)	2.66_a,c_ (1.83)	0.41_b_ (0.50)	10.87_a,c_ (9.84)	11.37_a,c_ (10.82)
Eating disordered attitudes	38		28.95_b_ (7.30)	5.03_b_ (1.31)	0.98_c_ (0.61)	10.11_b_ (12.83)	20.16_b_ (15.58)
ARFID	46		6.74_a_ (5.56)	3.42_c_ (1.92)	1.08_c_ (0.73)	16.50_b,c_ (12.92)	16.20_b,c_ (10.03)
One-way ANOVA		*F*(3) *η* ^*2*^	211.16* .62	21.19* .14	53.49* .29	12.02* .08	12.53* .09

Table abbreviations: *EAT-26* Eating Attitudes Test-26; *EDDS* Eating Disorder Diagnostic Scale, *EQoL* Eating-related Quality of Life impairment, *DASS-21* Depression, Stress, and Anxiety Scale-21, *OCI-R* Obsessive Compulsive Inventory-Revised

Means appearing in the same column with different subscripts are significantly different at the *p* < .05 level (Tamhane’s T2)

*p < .001

### Study aim 2: picky eating severity markers

Overall mean comparisons suggested that the four eating behavior groups differed significantly on all four variables (Tables 6 and 7). Identical patterns of results emerged for food neophobia and inflexible eating behaviors; typical eaters had significantly lower scores on both variables than any other eating group; picky eaters reported significantly higher scores than either typical eaters or participants with eating disordered attitudes. Participants reporting ARFID symptoms had significantly higher scores than any other eating group. All post-hoc differences were significant at the p < .001 level, with the exception of the difference between typical eaters and participants with disordered eating attitudes, who differed on eating inflexibility at *p* < .01 and on food neophobia at *p* < .05.

Post-hoc contrasts revealed that group difference on sensory sensitivity were less marked; typical eaters differed from the three other groups at the p < .001 level. Participants in the ARFID group scored significantly higher than picky eaters (*p =* .02), but neither group differed significantly from the disordered eating attitude group, whose mean score was between that of picky eaters and ARFID participants.

**Table 6:** Group differences and F-statistics for continuous picky eating variables

Eating behavior classification			Eating inflexibility (0–60)	Food neophobia (0–50)	Sensory sensitivity (0–5)
	*n*	Mean (SD)			
Typical eater	181		13.07_a_ (8.50)	12.59_a_ (8.39)	2.32_a_ (0.81)
Picky eater	132		30.70_b_ (13.92)	31.81_b_ (11.76)	2.82_b_ (0.89)
Eating disordered attitudes	37		19.27_c_ (9.48)	18.26_c_ (11.76)	3.11_b,c_ (0.74)
ARFID	46		42.98_d_ (11.51)	43.37_d_ (8.73)	3.26_c_ (0.84)
One-way ANOVA	413	*F*(4) *η* ^*2*^	122.32* .48	169.37* .56	15.91* .15

Means appearing in the same column with different subscripts are significantly different at the *p* < .05 level (Tamhane’s T2)

*p < .001

Finally, the four groups differed significantly on the proportion of participants who reported eating from a range of 20 or fewer foods: Cramer’s *V* = .50, *p* < .001. Exploratory post-hoc chi square tests with one degree of freedom were conducted to compare picky eaters to ARFID-only participants and to participants with disordered eating attitudes. A significantly higher proportion of ARFID participants compared to picky eaters reported eating from a range of 20 or fewer foods: *χ* ^*2*^(1) = 14.24, *ϕ* = .28, *p* < .001. The proportion of picky eaters who reported eating from a narrow range of foods was significantly higher than the proportion of participants with disordered eating attitudes, although the effect size was smaller: *χ* ^*2*^(1) = 4.6, *ϕ* = .16, *p* = .03.

**Table 7:** Narrow range eating behavior

Eating disorder classification	Number	Proportion eating from a range of 20 or fewer foods
Typical eater	189	9.0 %
Picky eater	133	42.86
Eating disordered attitudes	38	23.68
ARFID	46	73.91
*χ* ^*2*^(4)		95.08**

**significant at *p* < .001;, *p* < .001 (*χ* ^*2*^(1))

## Discussion

The present study is a first effort to understand the characteristics of the picky eating presentation of Avoidant/Restrictive Food Intake Disorder (ARFID) in a sample of community-dwelling adults. Self-reported picky eating was quite common, with 33 % of this MTurk sample expressing agreement with the statement “I am a picky eater.” ARFID symptoms were less common: 3.1 % of the MTurk sample (9.3 % of MTurk picky eaters) endorsed the presence of significant ARFID symptoms. Unsurprisingly, the support-group picky eaters were more likely than MTurk picky eaters to endorse symptoms of ARFID: 44.4 % of this sample (all of whom were picky eaters) reported experiencing ARFID symptoms due to their picky eating.

Replicating and extending findings by Wildes et al. [34]), we found that picky eating and symptoms of ARFID can exist independently of restricting disordered eating attitudes or behaviors in internet-using adults. Participants with ARFID endorsed comparable levels of eating-related quality of life impairment, internalizing distress, and OCD symptomatology to an established clinical analogue population, adults endorsing elevated disordered eating attitudes (Altman et al. [1]). We also showed that picky eating in the absence of ARFID symptoms does not appear to be associated with significant comorbidity, although picky eaters endorsed more impaired eating-related quality of life than typical eaters on a measure of interference and impairment related to eating behaviors. This finding adds to the existing literature on the relationship between adult picky eating and symptoms of OCD, depression, and social anxiety, demonstrating that, among internet using adults, picky eating does not appear to be associated with elevated symptoms of psychopathology unless it is severe enough to lead to symptoms of ARFID (e.g., [18]; Wildes et al. [34]).

In our second study aim, we attempted to identify markers of picky eating severity that can be used to differentiate picky eaters from individuals with other eating disturbances (e.g., disordered eating attitudes), and picky eaters with ARFID symptoms from those without. We explored four features of picky eating, selected based on prior phenomenological research on child and adult picky eating, that might differ between more and less severe picky eaters, and that we expected to differentiate picky eaters from both typical eaters and those endorsing disordered eating attitudes. Contrary to our hypothesis, based on previous findings in child and adult picky eaters (e.g., [7, 10, 18, 30]), degree of self-reported sensory sensitivity did not strongly differentiate picky eaters with or without ARFID from participants with disordered eating, nor did it differentiate picky eaters with ARFID symptoms from less severe picky eaters. Three other features of picky eating, rigid eating behaviors, food neophobia, and eating from a range of 20 or fewer foods, clearly differentiated picky eaters from typical eaters and those reporting eating disordered attitudes, and picky eaters with ARFID symptoms from those without. To our knowledge, our study is the first to identify potential markers of picky eating severity which may be useful in the assessment of picky eating and ARFID and in measuring outcomes in clinical practice and clinical trials for treatment of these feeding problems.

### Limitations

A major limitation of this study was that it relied on self-report. In addition, because ARFID is a new diagnosis and adult picky eating, while common, is very under-studied, we relied on author-developed questionnaires when previously validated questionnaires were not available to assess the constructs of interest (e.g., the presence of ARFID symptoms secondary to picky eating). Further evidence for the reliability and validity of this instrument from independent samples is needed, but our initial findings support its use to identify individuals with symptoms of ARFID in the internet-using population. Our findings suggest promising directions for future research, but should be replicated and extended using additional methodologies, including behavioral observation, diagnostic interviewing, and psychophysiological testing.

A second major limitation to the present study was the use of two distinct, and demographically different, samples. Because ARFID is a very low-baserate disorder, we chose to sample a group of participants who were more likely to experience the psychopathology of interest (e.g., support-seeking adult picky eaters). As is often the case, participants from this semi-clinical sample differed in several significant demographic ways from those in the unselected Mechanical Turk sample. Because of low power in the MTurk sample (just 3.1 % of participants endorsed ARFID symptoms in this sample) and because of the lack of typical eaters in the PEAS sample, it was not possible to replicate our major findings within either sample independently. This study is a preliminary exploration of a previously under-recognized population. Our findings should be interpreted with caution, and replicated in better-characterized and more representative samples.

Finally, this study focused on the presentation of ARFID characterized by picky eating. Our findings on symptom correlates and features of ARFID symptoms only apply to the picky eating presentation of ARFID; our study does not include ARFID symptoms due to limited appetite/interest in food or avoidance of eating because of fear of negative consequences. Future research should explore the prevalence of these three types of feeding disturbance in the adult population, and the degree to which they co-occur in the same individuals.

## Conclusions

This study is the first to explore the features of ARFID secondary to picky eating in an adult sample. Our findings demonstrate that ARFID symptoms are relatively common among internet-using adult picky eaters, and very common in adult picky eaters who seek support on the internet. Participants with ARFID symptoms experienced eating-related impairment, internalizing distress, and OCD symptoms at levels comparable to individuals with disordered eating attitudes, yet there is no evidence-based treatment for ARFID in adults and typically developing school-aged children, and very little treatment research has been conducted in these populations. In the 2006 National Eating Disorder QI Collaborative study, adolescents with ARFID who presented underweight were less likely than those with restricting eating disorders to achieve weight recovery, and more likely to drop out of family-based therapy and medical management, efficacious treatments for anorexia and bulimia nervosa that do not appear to be as acceptable or helpful to individuals with ARFID (e.g., [13]). There is evidence for the efficacy of intensive behavioral, applied behavior analysis-based treatments for feeding problems in chronologically or developmentally young children [20]. However, this treatment is not appropriate for typically developing adults or older children. There is a clear need for developmentally appropriate treatments for adults whose picky eating leads to significant impairment.

This study also introduced several novel instruments, including the ARFID symptom checklist used to identify picky eaters experiencing weight loss, nutritional deficiencies, nutritional supplement dependence, or psychosocial impairment. Although this instrument has not yet been used outside of the development sample, its ability to identify a group reporting significant comorbidity and impairment, and distinctive eating behaviors, supports its usefulness as a screening instrument for ARFID. In addition, our finding that picky eaters who report significant picky-eating related impairment also endorse higher levels of food neophobia and eating inflexibility, and are more likely to eat from a range of 20 or fewer foods, introduces several potential behavioral markers of picky eating severity, and identifies potential outcomes of interest in future studies of picky eating and ARFID treatment.

## Appendix

### Avoidant/restrictive food intake disorder self-report

“You said that you were a picky eater. Please answer the following questions about your PICKY EATING habits. If you have a medical condition that affects your diet, if you are a vegetarian or vegan, or if you do not eat certain foods for religious reasons, please ONLY consider the effect of your pickiness, not your other eating habits or restrictions. Has your PICKY EATING led to any of the following?”

0 = None 1 = Some 2 = Significant

1. Weight loss
2. Nutritional deficiency
3. Needing to take vitamins or supplements (pills or drinks like Ensure) to get adequate nutrition
4. Interference with academic/work performance
5. Interference with your social life
6. Interference with your relationship with your family

### Inflexibility index

0 = Never/Strongly disagree; 5 = Always/Strongly agree

1. I avoid letting different foods touch on my plate, even when they are both foods that I like
2. I prefer to eat with certain specific utensils or dishes.
3. If I dislike a food, I will not eat it under any circumstances
4. I do not like mixed foods (like stews or casseroles, or mixed vegetables)
5. I do not like foods with “things” in them (like raisins in cookies or nuts in bread)
6. I do not like sauces that have “lumps” in them (like bits of onion or chunks of tomato)
7. If a food that I usually like is not prepared in a specific way, I prefer not to eat it
8. The thought of eating a food I do not like fills me with anxiety
9. I am only comfortable eating in a certain place or places.
10. I prefer some brands of food to others (e.g., I will only eat one type of frozen fish sticks, or French fries from one fast food restaurant but not others)
11. If a food that I usually like touches a food that I don’t like, I prefer not to eat it
12. If a food I usually like is not presented in a certain way, I prefer not to eat it

### Sensory sensitivity

**Table:**

Not at all like me =0	Not like me =1	Not much like me =2	Somewhat like me =3	Like me =4	Just like me =5
1.My sense of taste is stronger/more sensitive than other people’s					
2. My sense of smell is stronger/more sensitive than other people’s					
3. I have trouble concentrating when I’m in a noisy environment					
4. I have trouble following a conversation when there are other conversations going on around me (like at a cocktail party or noisy restaurant)					
5. I am bothered by the smell of other people’s perfume or deodorant					
6. I notice new smells before other people do					
7. I am bothered by the sensation of seams or tags in my clothing against my skin					
8. I cannot wear certain fabrics because I find their texture against my skin uncomfortable					
9. I am very aware of the texture of food in my mouth					
10. I find loud noises (like fire alarms or sirens) very unpleasant					
11. I cannot concentrate on my work if I am listening to music					
